# Proximal third humeral shaft fractures fixed with long helical PHILOS plates in elderly patients: benefit of pre-contouring plates on a 3D-printed model—a retrospective study

**DOI:** 10.1186/s13018-018-0908-9

**Published:** 2018-08-17

**Authors:** Qiuke Wang, Jian Hu, Junjie Guan, Yunfeng Chen, Lei Wang

**Affiliations:** 1 Department of Orthopedic Surgery, Shanghai Jiao Tong University Affiliated Sixth People’s Hospital, 600 Yishan Road, Shanghai, 200233 People’s Republic of China; 2 Department of Pathology, Shanghai Eighth People’s Hospital, 8 Caobao Road, Shanghai, 200233 People’s Republic of China

**Keywords:** Helical plate, Humeral fracture, 3D-printed, MIPO

## Abstract

**Background:**

To explore the clinical efficacy of 3D printing fracture models to assist in creating pre-contoured plates to treat proximal third humeral shaft fractures.

**Methods:**

We retrospectively identified proximal third humeral shaft fractures treated between February 2012 and February 2015. The patients were divided into two groups according to the treatment procedure: a Synbone group and a 3D-printed group. In the Synbone group, long proximal humeral internal locking system plates were pre-contoured into helical shape on Synbones before surgery, while in the 3D-printed group, they were contoured on 3D-printed bone models. The pre-contoured plates were sterilized before surgery and were then used for fracture fixation during surgery. Duration of surgeries, blood loss volumes, the incidence of complications, and the time to fracture union were recorded, and functional outcomes were assessed by the Constant-Murley shoulder score and the Mayo Elbow Performance Score (MEPS) at 1-year follow-up.

**Results:**

The subjects comprised 46 patients; 25 patients were allocated to the Synbone group and the remaining 21 to the 3D-printed group. There was no significant difference between the baseline characteristics of the two groups. At the 1-year follow-up visit, all fractures were healed and showed a satisfactory outcome. There were no instances of iatrogenic radial nerve injury, and there was no significant difference between the two groups with regard to fracture union time, Constant-Murley score, or MEPS score. Surgery duration was significantly shorter in the 3D-printed group compared to the Synbone group (42.62 vs. 60.36 min, *P* = 0.001), and the 3D-printed group lost less blood during surgery (105.19 vs. 120.80 ml, *P* = 0.001). In addition, in the 3D-printed group, 9 surgeries were finished by senior attending doctors and 12 were finished by junior attending doctors; however, there was no significant difference between the 1-year outcomes of the two grades of surgeons.

**Conclusions:**

Our results show that the 3D printing technique is helpful in shortening the duration of surgery, reducing blood loss volume, and in making this surgical procedure easier for less-experienced surgeons.

**Trial registration:**

This clinical study was registered in CHICTR on September 30, 2017 (number 17012852).

## Background

Humeral shaft fractures account for approximately 3% of all bone fractures [1–4], and for most of these, non-operative treatment with a functional brace is recommended. However, the non-union rate of fractures in the proximal third of the humerus is relatively higher than for those in other regions with conservative treatment, and shoulder stiffness problems tend to appear after long-term external fixation [2, 5–7]. Moreover, clinical studies have reported that approximately 49.3% of proximal third humeral shaft fractures extend into the humeral head, which is difficult to verify on X-ray and should be treated with stable fixation [6]. Thus, surgery should be considered as an alternative plan for proximal third humeral shaft fractures.

The helical plating technique was first reported to be used in cases of proximal third humeral shaft non-union in 1999 [8]. This technique was developed and successfully used to treat proximal humeral shaft fractures [9–14]. The helical-shaped design is mainly aimed at minimizing the risk of radial nerve injury, because the plate roughly parallels the nerve from the proximal to the distal humerus. The locking plate is twisted approximately 90° to lie on the lateral aspect of the greater tuberosity proximally and the anterior or anteromedial aspect of the humeral shaft distally, so theoretically, most of the deltoid muscle attachment can be preserved and the radial nerve should not be entrapped in the distal approach. With concerns about soft tissue compromise, the minimally invasive plate osteosynthesis (MIPO) technique is considered to be an essential component of this surgery. In order to fix the fractures in the proximal humerus and the proximal third of the shaft simultaneously, we recommend long helical proximal humeral internal locking system (PHILOS) plate (DePuy Synthes, Zuchwil, Switzerland) rather than normal metaphyseal locking plate, and its safety and efficacy have been proven in our previous study [11].

Although the outcomes of this novel technique are satisfactory, we found it difficult to pre-contour a PHILOS plate for personalized application. Even though we molded the plate on a Synbone (Synbone AG, Malans, Switzerland) before surgery, it was always necessary to adjust its shape during the operation, which wasted much time, especially in the case of elderly patients. Old patients, in whom fractures were likely to be combined with severe osteoporosis, in our country are more likely to have a much shorter humerus than the standard Synbone. Consequently, we considered that a personalized 3D-printed model would be helpful in producing a better matched long helical PHILOS plate before surgery.

The purpose of this study was to investigate the benefit of pre-contouring long helical PHILOS plates on 3D-printed models for the treatment of proximal third humeral shaft fractures in elderly patients. We hypothesized that this individualized treatment plan would reduce the technical challenge, shorten the duration of surgery, and reduce blood loss volume.

## Methods

### Patients

We retrospectively identified patients, treated with long helical PHILOS plates between February 2012 and February 2015, from our trauma center’s database. Inclusion criteria were (I) aged 65 years or above, (II) presence of proximal third humeral shaft fractures (unilateral), (III) treated by the MIPO technique with a long helical PHILOS plate, and (IV) more than 12 months follow-up after surgery with complete follow-up data. Exclusion criteria included pathological fractures, open fractures, multiple fractures, or the presence of other diseases affecting the same upper limb.

Ultimately, 46 patients were included, and data regarding their medical histories, functional evaluations, and regular radiographic examinations were collected. All fractures were classified according to the AO/ASIF classification system [15]. Medical history information included gender, age, and surgical information (plate pre-contoured on a Synbone or 3D-printed model, the name of the surgeon, duration of surgery, and blood loss volume during surgery).

### 3D-printed model and plate preparation

A bilateral humerus CT scan (1-mm layer interval) was taken from patients in the 3D-printed group, and CT data was extracted for constructing the 3D-printed model, using medical 3D processing software (Mimics 16.0, Materialise, Belgium). The scapula, clavicle, and relative soft tissue were separated. A mirror image of the intact side was used to simulate the other side, and the models were printed using a 3D-printing machine (Lite, RS6000, UnionTech, Shanghai, China) with ultraviolet rays (UV) curable resin material. Total preparation time of a 3D bone model was approximately 3 h as long as the CT data is available.

**Fig. 1:**
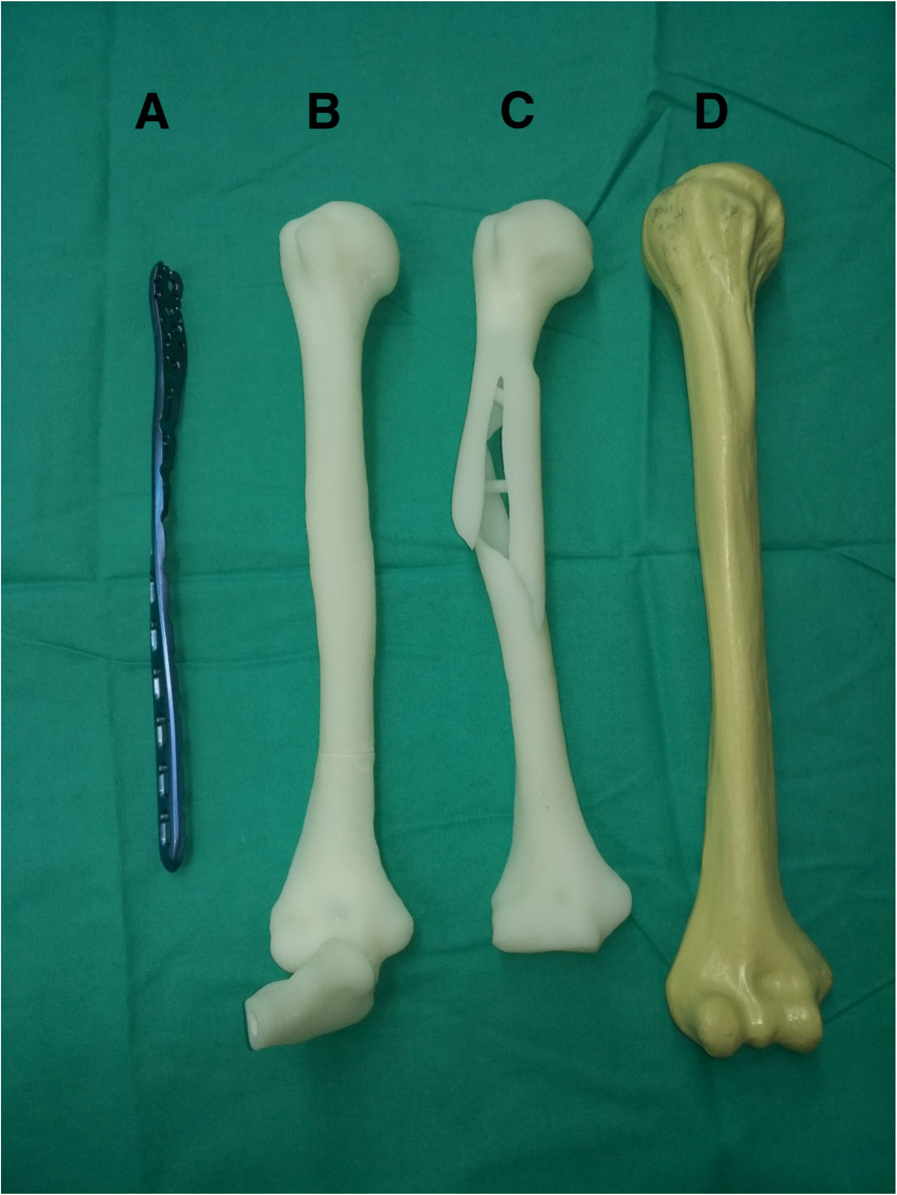
Pre-operative preparation of an old female patient. (**a**) A long helical PHILOS plate was pre-contoured on an intact 3D-printed model (**b**). (**b**) An intact 3D-printed model which was constructed by the mirror image of the contralateral side. (**c**) A 3D-printed model of the fractured bone. (**d**) A Synbone model

In the 3D-printed group, a long PHILOS plate was pre-contoured on an intact 3D-printed model, to ensure that it could be used on the fractured bone. In the Synbone group, the plate was pre-contoured on a standard Synbone model. The plate was twisted at approximately 60–90° (starting at the superior part of the humeral deltoid tuberosity), while the proximal part of the plate was located on the lateral side of the greater tubercle and the distal part on the anterior side of the distal humerus. Then, the contoured helical plate was sterilized on the day before the surgery. The 3D-printed models and the helical plate used in an elderly Asian female patient are shown in Fig. 1, and it is obvious that the true humerus is much shorter than the Synbone.

### Surgical procedure

**Fig. 2:**
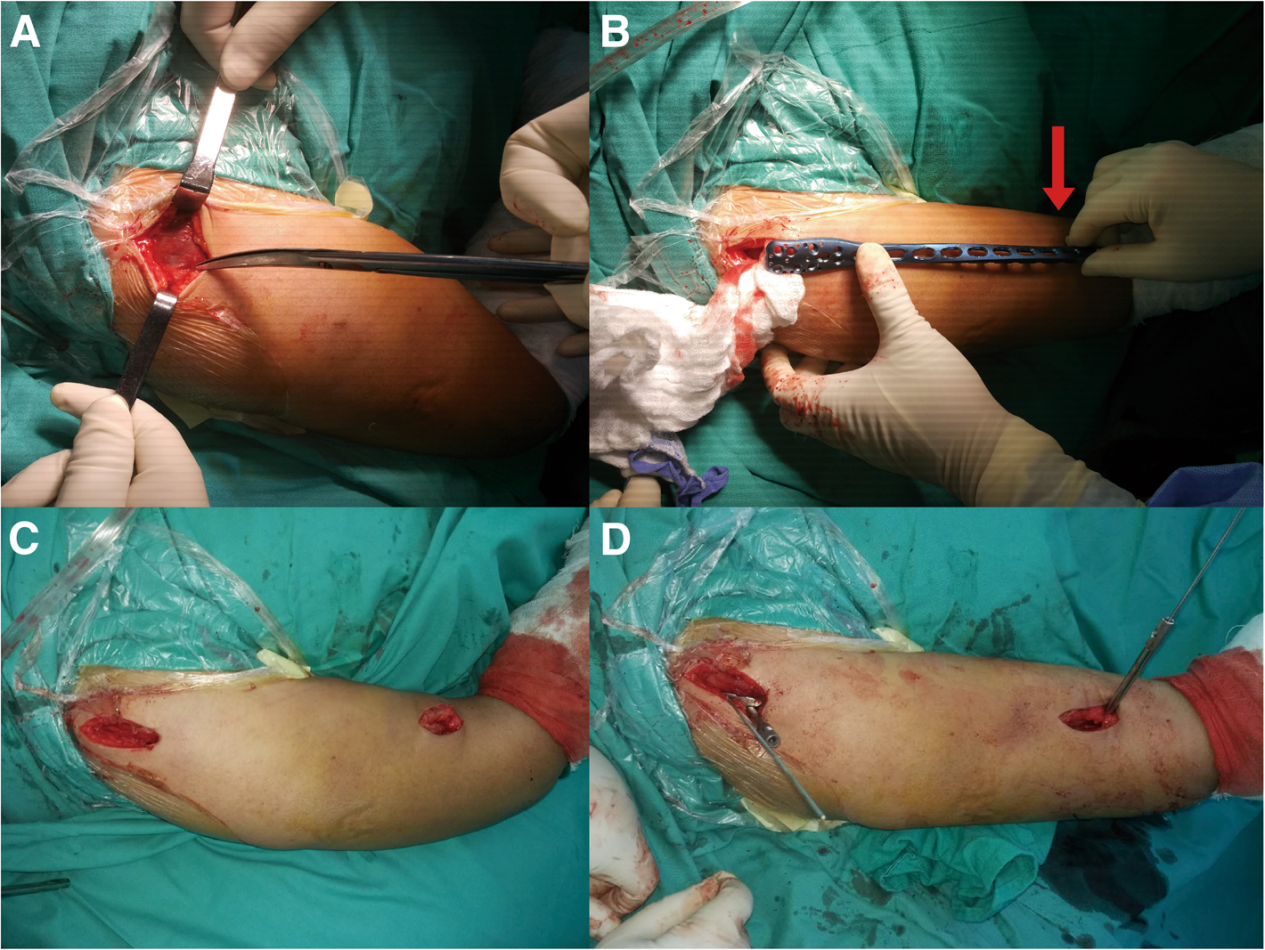
Surgical procedure. **a** An anterolateral acromial approach (ALA) was performed with a 5-cm skin incision proximally. **b** The site of distal approach was decided according to the length of the pre-contoured plate (red arrow). **c** Two approaches were made. **d** An extraperiosteal tunnel was made to connect both approaches, and the plate was placed with MIPO technique

The basic surgical steps were similar for the two groups and were as described in our previous study [11]. After general anesthesia or brachial plexus block anesthesia, the patient was placed in the beach-chair position, with the upper limb in full supination, and an anterolateral acromial approach (ALA) was performed with a 5-cm skin incision proximally, while the anterior approach was performed with a 5-cm skin incision distally by splitting the brachialis longitudinally just along the lateral side of the biceps brachii. The site of the distal approach was decided according to the length of the pre-contoured plate. During the distal anterior approach, care was taken not to injure the musculocutaneous nerve, which lies between the biceps and the brachialis; the dissection through the brachialis was performed bluntly but gently, without routine nerve exposure. An extraperiosteal tunnel was made to connect with both approaches, from the lateral part of the greater tubercle proximally to the anterior part of the humerus distally. The key point was that, in order to protect the deltoid attachment point, the tunnel did not pass through the humeral deltoid tuberosity but across it anteriorly. Using the MIPO technique, the pre-contoured helical long PHILOS plate was then inserted from the proximal approach, passed through the tunnel distally, and fixed on the humerus with locking screws through the ALA approach proximally (five or more screws inserted). According to AO principles, at least two or three screw holes should be left open over the fracture to decrease stress concentration [15]. So, usually, three to four screws were inserted distally according to the different fracture types (Fig. 2). Finally, intraoperative fluoroscopy was used to ensure a correct fracture reduction and positioning of the plate. Some adjustments were required if the fluoroscopy results were unsatisfactory. The name of the surgeon, duration of surgery (from incision to skin closure), and blood loss volume were recorded.

### Rehabilitation and follow-up

After surgery, all patients were recommended the same rehabilitation plan. They were allowed to perform passive range of motion exercises immediately, and active exercises were allowed after 2 weeks. Patients were recommended to attend follow-up visits at 4 weeks, 12 weeks, 6 months, and 12 months after surgery. At each follow-up visit, a regular X-ray examination was taken, and at the 12-month follow-up, functional evaluation was added.

### Statistical analysis

Categorical variables in each group are presented as count (percentage), and continuous variables are presented as mean ± standard deviation. All categorical variables were compared directly to each other using the chi-square test or Fisher’s exact tests. For continuous variables, Student’s two-tailed *t* tests were conducted. Results were considered significant when *P* < 0.05. The analysis was performed using the statistical program SPSS version 21 (IBM Corp., Armonk, NY, USA).

## Results

Among the 46 enrolled patients, 25 patients were treated with plates pre-contoured on standard Synbone before surgery, and the remaining 21 were contoured on 3D-printed models. Since we chose older patients to form our study population, the mean age of all patients was 71.45 years: 71.84 years in the Synbone group and 71.00 years in the 3D-printed group. The Synbone group consisted of 7 males and 18 females; 40% of these patients presented with a fracture that extended to the proximal humerus, and the mean follow-up time was 18.48 months. Twenty-one patients were allocated to the 3D-printed group, 7 were males and 14 were females; in 42.9% of cases, the proximal humerus was involved, and the mean follow-up time was 16.95 months. The two groups were considered homogeneous since there were no significant differences between these baseline characteristics (Table 1).

**Table 1:** Baseline characteristics of the Synbone group and 3D-printed group

	Synbone group	3D-printed group	*P* value
Age	71.84 ± 4.81	71.00 ± 5.81	0.594
Gender			0.695
Male	7 (28%)	7 (33.3%)	
Female	18 (72%)	14 (66.7%)	
Proximal humerus involved			0.845
Yes	10 (40%)	9 (42.9%)	
No	15 (60%)	12 (57.1%)	
Fracture type (AO/OTA)			0.782
A	4 (8.7%)	3 (6.5%)	
B	13 (28.3%)	13 (28.3%)	
C	8 (17.4%)	5 (10.9%)	
Follow-up months	18.48 ± 6.25	16.95 ± 5.12	0.367

**Fig. 3:**
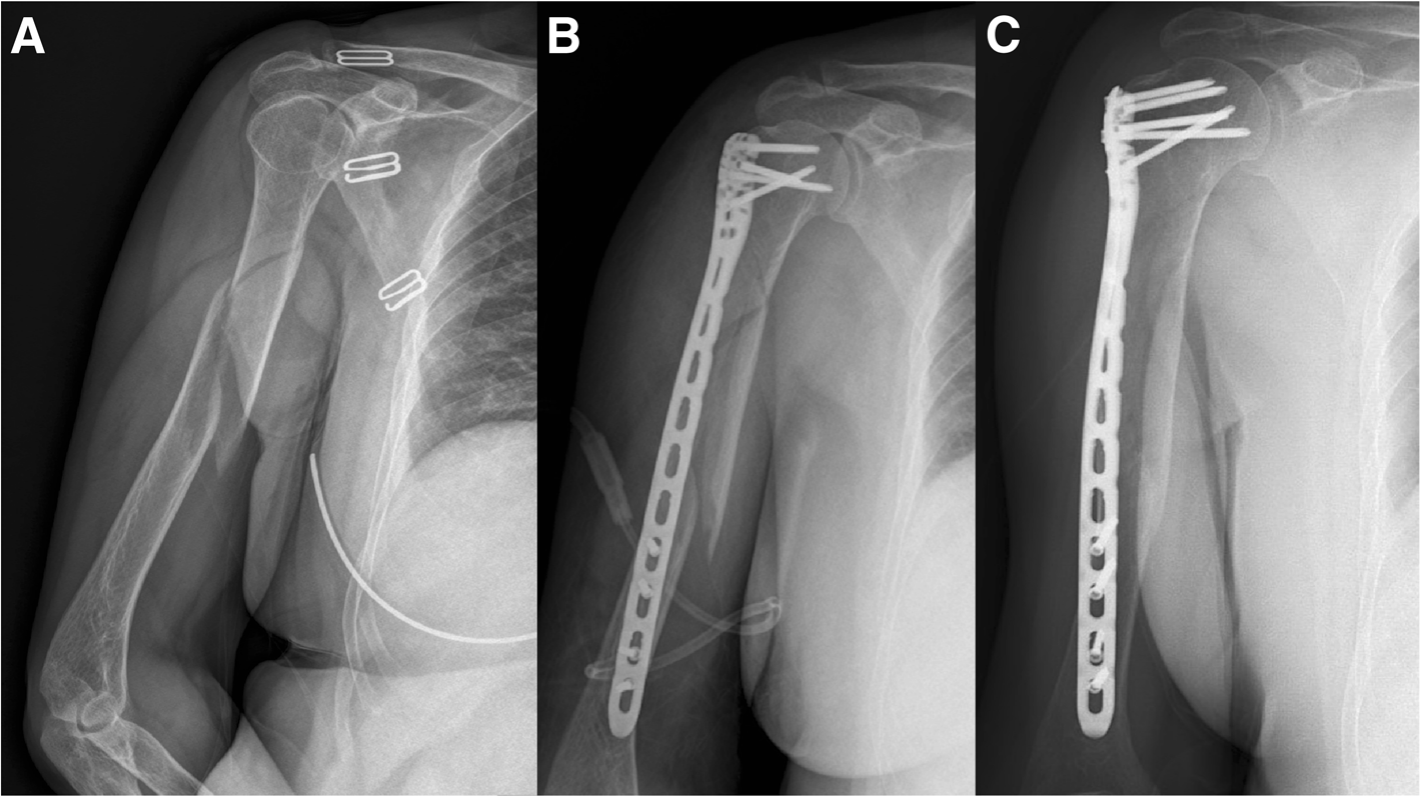
A patient, suffered proximal third humeral shaft fracture from a simple fall, was allocated to the 3D-printed group. **a** Pre-operative X-ray examination. **b** The first day after surgery. **c** The completely healed fracture at 1-year follow-up

At the 1-year follow-up visit, all fractures were healed and showed a satisfactory outcome with respect to the radiographic examinations (Fig. 2). There were no significant differences between the two groups with regard to the fracture union time, Constant-Murley score or Mayo Elbow Performance Score (MEPS) (Table 2). One patient in the Synbone group suffered shoulder impingement, and another one in the 3D-printed group presented with a superficial infection after surgery (Fig. 3). There were no instances of iatrogenic radial nerve injury or other major complications.

**Table 2:** Outcomes of the Synbone group and 3D-printed group

	Synbone group	3D-printed group	*P* value
Time to fracture union (weeks)	16.16 ± 3.65	15.70 ± 2.96	0.976
Constant-Murley score	76.80 ± 6.67	76.95 ± 6.03	0.936
MEPS score	96.80 ± 3.79	94.32 ± 4.02	0.928
Duration of surgery (min)	60.36 ± 10.20	42.62 ± 7.61	0.001*
Blood loss volume (ml)	120.80 ± 10.61	105.19 ± 14.67	0.001*
Surgeon			
Senior attending doctor	25	9	
Junior attending doctor	0	12	

*Differences are statistically significant

All surgeries in the Synbone group were finished by senior attending doctors, while in the 3D-printed group, 9 surgeries were finished by senior attending doctors and 12 were finished by junior attending doctors. The duration of surgery was significantly shorter in the 3D-printed group than in the Synbone group (42.62 vs. 60.36, *P* = 0.001). In addition, patients in the 3D-printed group lost significantly less blood during surgery (105.19 vs. 120.80 ml, *P* = 0.001) (Table 2). In the 3D-printed group, there were no significant differences between the baseline characteristics of the two grades of surgeons. However, we also found no significant difference between the outcomes (Table 3).

**Table 3:** Outcomes of the two grades of surgeons of the 3D-printed group

	Senior attending doctor	Junior attending doctor	*P* value
Age	70.56 ± 6.02	71.33 ± 5.88	0.770
Gender (male/female)	2/7	5/7	0.642
Fracture type (A/B/C)	2/4/3	1/9/2	0.355
Proximal humerus involved (yes/no)	5/4	7/5	0.899
Time to fracture union (weeks)	15.78 ± 2.11	16.50 ± 3.53	0.593
Constant-Murley score	77.33 ± 6.75	76.67 ± 5.74	0.809
MEPS score	97.22 ± 2.63	96.67 ± 4.92	0.763
Duration of surgery (min)	39.89 ± 8.07	44.67 ± 6.88	0.160
Blood loss volume (ml)	107.00 ± 18.01	103.83 ± 12.26	0.637

## Discussion

The optimal treatment for humeral shaft fracture remains controversial. Although a large proportion of these fractures can be treated without surgery, a recent study, involving a randomized controlled trial, compared bridge plate with functional brace fixation for humeral shaft fractures and concluded that surgical plating has a statistically significant advantage with a better DASH score, lower non-union rate, and lower residual deformity rate [1]. As for proximal third humeral shaft fractures, they were thought to be complicated with a higher non-union rate when treated conservatively compared with middle and distal fractures [2, 5, 16]. Since the helical plating technique was introduced for the treatment of humeral fractures, some studies have shown that this technique resulted in increased stiffness compared to fixation with a straight plate under torsional loading and produced satisfactory clinical outcomes [14, 17]. However, how to produce a suitable helical plate for each individual patient is a big question for surgeons. Previous studies have proven that the 3D printing technique is a good tool for designing surgical plans and pre-contouring plates used to treat other bone fractures [18–20]. Our results demonstrate the benefit of pre-contouring plates on a 3D-printed model for this special technique.

In this study, all kinds of fractures (from type A to type C) were treated by helical plating technique, and satisfactory outcomes were obtained. It was coincident with our previous cadaveric study results [11], so we thought this special technique was a good choice for these fractures. Previously, Stedtfeld and Biber reported that approximately 49.3% of the proximal third humeral shaft fractures extend into the humeral head and that this type of fracture cannot be characterized by conventional AO classification [6]. In our study, a total of 41.3% (19/46) of fractures involved the proximal humerus, a rate slightly lower compared with their report, but still a high rate of these fractures. Consequently, attention should be paid on the proximal third humeral shaft fractures since about half of them need adequate proximal fixation.

At the 1-year follow-up visit, all fractures were healed and none of the patients had suffered non-union, an outcome better than that reported for other treatment methods [1, 2, 5, 9, 21–23]. The mean union times of the Synbone group and the 3D-printed groups were 16.16 and 15.57 weeks, respectively, which was similar to other studies even though our patients were older than in other studies [11, 17, 24]. Functional evaluations were satisfactory but were worse than those reported by others who conducted the same surgeries (Constant-Murley score 76.80, 76.95 vs. 88.6) [13, 17]. This may be attributed to the fact that our population was much older, so that humeral fracture might be combined with rotator cuff degeneration in our enrolled patients.

The primary outcomes of this study were that surgical duration and blood loss were reduced by the use of a 3D-printed model for pre-contouring the plates before surgery. This result was consistent with our hypothesis and can be explained by the fact that the humeri of older patients in our country are much shorter than the standard Synbone, requiring surgeons to adjust the plates during surgery. Since the 3D-printed model represented the actual size of the bone, the plates pre-contoured on these models were always suitable for fixing the fractures. Because of MIPO technique application, there was only 15 ml of blood loss difference between the two groups; maybe it was not clinically relevant, but on the whole, it reduced 12.5% of blood loss volume and presented a small part of the benefit of 3D-printed technique.

We compared the outcomes between the two grades of surgeons in the 3D-printed group. Although senior attending doctors are much more experienced than junior attending doctors, the results showed that there was no significant difference between them in terms of outcome. We believed that the 3D printing technique would make this novel technique much easier and make it available for use by less specialized surgeons. However, since all fractures in the Synbone group were finished by senior attending doctors, it was impossible to compare the results with a control group.

There are some limitations to this study: (I) the retrospective design limits the level of evidence and only represents one single center; (II) some patients who died within 1 year of surgery are excluded from this study, which may influence the final results; (III) all these surgeries were finished by surgeons in one trauma center, so personal differences cannot be avoided; and (IV) this study only included Asian population, and maybe the results could be challenged by other races because of different skeletal sizes.

## Conclusions

Our results demonstrated that pre-contouring plates into a helical shape on a 3D-printed model was helpful in shortening the duration of surgery and reducing blood loss volume. In addition, the 3D printing technique could make this surgical procedure easier, enabling a widespread application for the treatment of proximal third humeral shaft fractures.

## Abbreviations

3D print: Three-dimensional print
MIPO: Minimally invasive plate osteosynthesis
UV: Ultraviolet rays
